# Characterization of the complete mitochondrial genome of the moss, *Myurella julacea* (Schwägr.) Schimp. (Bryidae, Theliaceae)

**DOI:** 10.1080/23802359.2021.1903354

**Published:** 2021-03-28

**Authors:** Jin-Woo Jung, Yeong-Deok Han, Young-Jun Yoon

**Affiliations:** Research Center for Endangered Species, National Institute of Ecology, Yeongyang-Gun, Korea

**Keywords:** *Myurella julacea*, complete mitochondrial genome, moss

## Abstract

The present study reports the complete mitochondrial genome of *Myurella julacea* (Schwägr.) Schimp. (GenBank accession number MT850126); the genome size was 104,979 bp. The gene arrangement was found to be similar to that in other bryophytes, and the genome consisted of 40 protein-coding genes (PCGs), 3 ribosomal RNAs (rRNAs), and 24 transfer RNAs (tRNAs). The phylogenic relationship was analyzed by construction of a phylogenetic tree based on the mitogenome of *M*. *julacea* and 25 other bryophytes publicly available in GenBank. The complete mitogenome of *M. julacea* is expected to provide insights into the evolution of species belonging to the order Hypnales.

In South Korea, the incidence of *M. julacea* was noted only in Jeongseon-gun of the Gangwon province by the corresponding author. This minute moss has shoots that are only about 1–3 cm long and less than 0.5 mm wide. Fresh shoots are smooth, cylindrical, wiry, branched, and silvery-blue-green in color. It is known to grow in rock crevices at calcareous sites (Noguchi 1991). To date, 4 *Myurella* species have been reported (Ignatov and Kuznetsova 2011), out of which 3 (*M. julacea*, *M. sibirica*, *and M. tenerrima*) have been found in neighboring nations such as China, Japan, and Far East Russia (Noguchi 1991; Wu et al. 2002).

Despite its widespread distribution in the northern hemisphere (Higuchi et al. 2012), *M. julacea* is classified as a Threatened Species (under Critically Endangered and Endangered categories) in the Red Data Book of Japan for plants by the Environment Agency due to a high possibility of extinction faced by this plant (Higuchi and Arikawa 2006). Small habitat range and habitat loss from rapid environmental changes, such as deforestation and development of limestone mines, have driven this species toward extinction. Since *M. julacea* encounters similar threats in South Korea, we are currently reviewing its conservation status for potential inclusion in the list of Endangered Species in South Korea.

In this study, *M. julacea* specimens for complete mitogenome sequencing were collected from a naturally growing population in a 5 × 5 cm patch in Jeongseon-gun, Gangwon-do, South Korea (37°27′5.9′′N, 128°41′5′′E) on 21 December, 2019. The specimen was deposited at the Jeonbuk National University (JNU Herbarium) in Korea with the accession number PSJ-17123151. Purified genomic DNA was extracted from a portion of the specimen using a DNeasy^®^ Plant Mini Kit (Qiagen, Hilden, Germany). A DNA library was then prepared from 550 bp fragments and read by 150-bp paired-end (PE) sequencing using the Illumina HiSeq sequencing platform (Illumina, San Diego, CA). Reads were filtered, and the partial sequence of NADH dehydrogenase subunit 5 of *M*. *julacea* (GenBank accession number MG030707) used as the seed sequence for the seed-and-extend algorithm using NOVOPlasty 2.4 software (Dierckxsens et al. 2016). Gene annotation was performed by comparison with the mitogenome of *Climacium dendroides* (GenBank accession number MN942036) using the Geneious 8.1.9 software.

The mitochondrial genome of *M. julacea* had a size of 104,979 bp and was a circular molecule consisting of 40 PCGs, 24 tRNAs, and 3 rRNAs. The overall nucleotide composition was 29.5% A, 29.4% T, 19.8% C, and 21.3% G; and the A + T content was calculated to be 58.9%. The gene arrangement was similar to that of other conventional Bryophyta mitogenomes.

The phylogenetic relationship of *M. julacea* was explored using a maximum likelihood (ML) analysis of the concatenated alignment of 33 PCGs corresponding to 22 Bryophyta mitogenomes and 3 outgroup Marchantiophyta ones (Figure 1). ML analysis was performed using the JTT matrix-based model based on 1000 bootstrap replicates with the MEGA X software. The complete mitochondrial genome of *M. julacea* could function as a reference for developing markers for further studies on the phylogeny and evolution of the *Myurella* genus. In addition, information on the *M. julacea* mitochondrial genome would be useful for species identification or confirmation.

**Figure 1.**
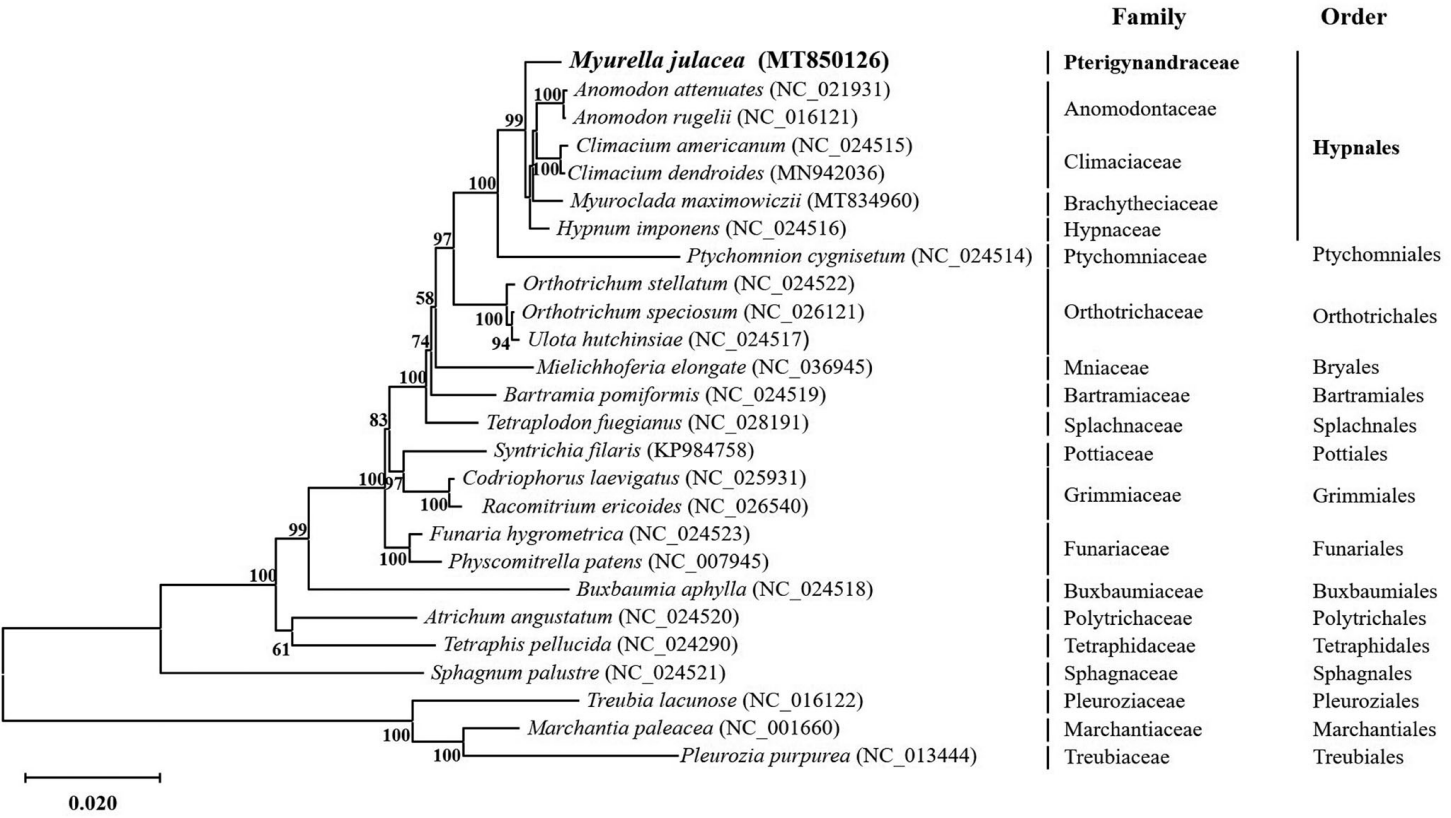
Maximum likelihood phylogenetic tree constructed from 33 mitochondrial protein-coding genes from *Myurella julacea* and 25 other bryophytes. A bootstrap value is displayed on each node when it is above 50%. Three Marchantiophyta species were selected as outgroups.

## Data Availability

Mitochondrial genome sequence can be accessed via accession number MT850126 in GenBank of NCBI at https://www.ncbi.nlm.nih.gov. The associated BioProject, SRA, and Bio-Sample numbers are PRJNA697075, SAMN17638144, and SRR13576430, respectively.
